# Identification of Digestive Enzyme Inhibitors from* Ludwigia octovalvis* (Jacq.) P.H.Raven

**DOI:** 10.1155/2018/8781352

**Published:** 2018-07-16

**Authors:** Dulce Morales, Guillermo Ramirez, Armando Herrera-Arellano, Jaime Tortoriello, Miguel Zavala, Alejandro Zamilpa

**Affiliations:** ^1^Facultad de Medicina, Universidad Autónoma del Estado de Morelos, Cuernavaca 62350, Mexico; ^2^Centro de Investigación Biomédica del Sur, Instituto Mexicano del Seguro Social, Xochitepec 62790, Mexico; ^3^Departamento de Sistemas Biológicos, UAM–Xochimilco, Mexico City 04960, Mexico

## Abstract

Current antiobesity and antidiabetic tools have been insufficient to curb these diseases and frequently cause side effects; therefore, new pancreatic lipase and *α*–glucosidase inhibitors could be excellent aids for the prevention and treatment of these diseases. The aim of this study was to identify, quantify, and characterize the chemical compounds with the highest degree of inhibitory activity of these enzymes, contained in a* Ludwigia octovalvis* hydroalcoholic extract. Chemical purification was performed by liquid–liquid separation and column chromatography. Inhibitory activities were measured* in vitro*, employing acarbose, orlistat, and a* Camellia sinensis* hydroalcoholic extract as references. For structural elucidation, Nuclear Magnetic Resonance was carried out, and High Performance Liquid Chromatography was used to quantify the compounds. For *α*–glucosidases,* L. octovalvis* hydroalcoholic extract and its ethyl acetate fraction showed half–maximal Inhibitory Concentration (IC_50_) values of 700 and 250 *μ*g/mL, for lipase, 480 and 718 *μ*g/mL, while* C. sinensis* showed 260 and 587 *μ*g/mL. The most active compounds were identified as ethyl gallate (**1**, IC_50_ 832 *μ*M) and gallic acid (**2**, IC_50_ 969 *μ*M); both displayed competitive inhibition of *α*–glucosidases and isoorientin (**3**, IC_50_ 201 *μ*M), which displayed uncompetitive inhibition of lipase. These data could be useful in the development of a novel phytopharmaceutical drug.

## 1. Introduction

Although *α*–glucosidase inhibitors such as acarbose and pancreatic lipase inhibitors such as orlistat are one of the safest antiobesity and antidiabetic drugs for weight loss and regulation of several metabolic and cardiovascular parameters in adults [1–3], these drugs have unpleasant gastrointestinal side effects that frequently result in therapy abandonment [4]. Therefore, it is necessary to continue the search for new alternatives to *α*–glucosidase and pancreatic lipase inhibitors, with milder side effects and which contribute to the treatment of obesity and type 2 diabetes mellitus, in conjunction with current therapies.

Treatment with acarbose brings forth benefits in the regulation of HbA1c, blood pressure, coagulation factors, thickness of the intimal layer of the carotid, endothelial dysfunction, serum glucose, and postprandial insulin [2], being especially useful in the treatment of diabetic patients with adequate baseline control but persistent postprandial hyperglycaemia [1]. While orlistat treatment not only produces a reduction in body weight and waist diameter, it also decreases HbA1c, blood pressure, and cholesterol [5], reducing the incidence of type 2 diabetes mellitus. In addition, orlistat is currently the only drug approved by the Food and Drug Administration (FDA) for the treatment of obesity in children [3].

*Ludwigia octovalvis *(Jacq.) P.H.Raven (Onagraceae) [syn:* Jussiaea suffruticosa* L.,* Jussiaea pubescens* L., and* Jussiaea angustifolia* Lamk] is an helophyte, erect, herb with oblong–lanceolate leaves and solitary flowers of four yellow petals [6]. According to Mexican data, this species is not on a protection status [7]. Almost all parts of the plant have been reported as having several medicinal uses [8, 9], among them, the antidiabetic use by Mexican and Indian healers [10, 11], in which the boiled extract or the juice of the whole plant is used. Previous phytochemical studies have described the presence of flavonoids, phenolic acids, polyphenols, saponins, sterols, tannins, and triterpenoids [12–15] in different organs of this medicinal plant. Several pharmacological effects such as hypoglycaemic [8], antihyperglycaemic [16, 17], and antiproliferative, in 3T3–L1 adipocytes [18], have been described through various models. Moreover, the hydroalcoholic extract of* L. octovalvis* leaves was the most effective in the inhibition of *α*–glucosidases and pancreatic lipase in a screening of 23 extracts of medicinal plants reported as traditional treatments for type 2 diabetes mellitus [10]. In addition, a report also exists on* L. octovalvis* antidiarrheal activity, probably mediated by regulation of gastrointestinal motility [19]; this activity could help reduce some of the side effects of intestinal enzyme inhibition, such as faecal urgency or abdominal pain.

The aim of this work was to isolate, identify, quantify, and characterize the compounds with the greatest inhibitory activity of *α*–glucosidases and pancreatic lipase, in the hydroalcoholic extract of* L. octovalvis* leaves, through its bioassay–guided fractionation.

## 2. Materials and Methods

### 2.1. General

All chemicals were of analytical–reagent grade. Corn starch (S4126); 2,3–dimercapto–1–propanol tributyrate (DMPTB 97%, 282413); 5,5′–dithiobis(2–nitrobenzoic acid) (DTNB ≥98%, D8130); lipase from porcine pancreas (PPL type II, 100–500 units/mg, L3126); Triton X–100 (X100); SDS (≥98.5%, L3771); glycerol (≥99.5%, GE17–1325–01); DMSO (≥99.9%, 547239); polyethylene glycol (PEG, 1546580); 2–aminoethyl diphenylborinate (97%, D9754); isoorientin (≥98%; I1536); and gallic acid (≥97%, 27645) were purchased from Sigma–Aldrich (St. Louis, MO). Miscellaneous solvents were purchased from Merck KGaA (Darmstadt, Germany).

Orlistat (Lysthin, PsicoFarma, Mexico City) and acarbose (Sincrosa, Alpharma, Mexico City) were purified by silica chromatography and crystallized, to be used as positive controls for enzyme inhibition assays.

Thin layer chromatography (TLC) was performed using silica gel 60 RP–18 F254s aluminium sheets (105560, Merck KGaA). TLC plates were analysed under UV light at 254 and 360 nm, using the Natural Products–PEG reagent (NP–PEG; 1% methanolic solution of diphenylboryloxyethylamine followed by 5% ethanolic PEG) as chemical detection system [20].

Melting points were obtained on a Thermo Scientific IA9000 series melting point apparatus (Electrothermal, Essex, UK).

Nuclear Magnetic Resonance (NMR) ^1^H (400 MHz) and NMR ^13^C (100 MHz) spectra were obtained with Varian INOVA–400 equipment (Varian Co., Palo Alto, CA) using tetramethylsilane as internal standard.

### 2.2. Plant Material and Preparation of Extracts

Leaves of* L. octovalvis* were collected at Xochitepec, Morelos, Mexico (18°47′40.70^″^ N, 99°11′49.27^″^ W), between September and October of 2012. A voucher of plant material was deposited under code number 34667 at the HUMO Herbarium in the* Centro de Investigación en Biodiversidad y Conservación* of the Autonomous University of the State of Morelos (*Universidad Autónoma del Estado de Morelos*–*CIByC*–*UAEM*, Morelos, Mexico).

*Camellia sinensis* (L.) Kuntze (Theaceae) commercial ground leaves, purchased at a Japanese specialty store (Yamamotoyama, Pomona, CA), was used as a positive vegetal control. Plant names were checked and updated with the online website http://www.theplantlist.org. [21].

Fresh leaves of* L. octovalvis* were washed and dried under dark conditions at room temperature and then milled to 4–6 mm. Ground material (1 kg) was extracted (1:10 ratio, w/v) with a 60% ethanol aqueous solution at 25°C for 24 h. The liquid extract was paper-filtered, concentrated in a rotary evaporator Laborota 4000 (Heidolph, Schwabach, Germany) under reduced pressure at 50°C, and freeze-dried to obtain 337 g of brown powder (32.4% yield). This dry extract (LoHAE) was stored at 4°C until its pharmacological and phytochemical analysis.* C. sinensis* hydroalcoholic extract (CsHAE) was identically prepared.

### 2.3. Fractionation of LoHAE and Purification of Active Fractions

One hundred and ninety grams of LoHAE was subjected to a liquid–liquid separation process using water and ethyl acetate. The solvent of both fractions was eliminated by low pressure distillation to obtain an organic fraction (LoEAF) and an aqueous fraction (LoAqF).

The less polar fraction (LoEAF, 25 g) was subjected to a chromatographic silica gel 60 column (109385, Merck KGaA) using dichloromethane/methanol gradient system as mobile phase, to give 69 samples of 150 mL each. The separation process was monitored by TLC and all the samples were grouped into 20 final fractions. The most representative fractions (yields ≥5%; C1F1–C1F6) were subjected to both assays.

The active fractions C1F4 and C1F6 were fractionated using column chromatography with silica gel LiChroprep® RP–18 (113900, Merck KGaA) and a mixture of water/acetonitrile. All the fractions were analysed by TLC and the samples with similar chemical composition were grouped.

From C1F4 (186 mg), 10 final fractions were obtained, of which C2F1 produced a white precipitate, which was found to be a pure compound by TLC and High Performance Liquid Chromatography (HPLC).

**Scheme 1:**
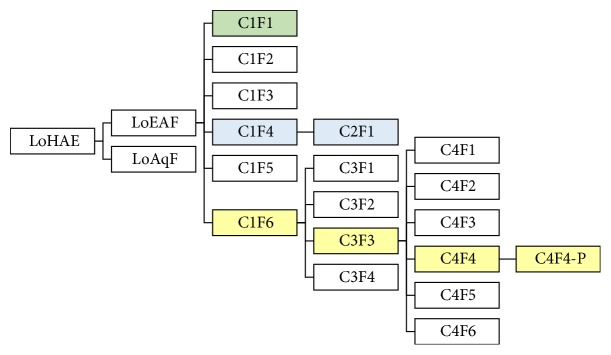
Fractionation of* L. octovalvis* hydroalcoholic extract (LoHAE). The isolation process of the active compounds is illustrated by colors: green for ethyl gallate, blue for gallic acid, and yellow for isoorientin.

From C1F6 (1.1 g), 19 final fractions were obtained; the most representative (yields ≥5%) were C3F1, C3F2, C3F3, and C3F4. Fraction C3F3 was purified, obtaining fractions C4F1, C4F2, C4F3, C4F4, C4F5, and C4F6. Fraction C4F4 produced an orange/yellow precipitate (C4F4–P, 12 mg). All these fractions (see Scheme 1) were subjected to the pharmacological assay.

### 2.4. HPLC Analysis

HPLC analysis was performed on a chromatographic system equipped with a Waters Alliance Separation Module (2695, Waters Corporation, Milford, MA) and a photodiode array detector (2996, Waters Corporation), employing Empower Pro software (Waters Corporation). Separation was carried out using a Supelcosil LC–F HPLC column (59158, Supelco, Bellefonte, PA). The mobile phase consisted of a mixture of trifluoroacetic acid solution (solvent A, 0.5%) and acetonitrile (solvent B) with the following ratios: A:B = 100:0 (0–1 min); 95:5 (2–3 min); 70:30 (4–7 min); 50:50 (8–22 min); 20:80 (23 min); 0:100 (24–26 min); 100:0 (27–30 min). The sample injection volume was 10 mL with a 0.9 mL/min flow rate during 30 min. The detection wavelength was 190–600 nm.

Quantification of the isolated compounds was achieved using calibration curves and LoHAE or LoEAF HPLC analysis. The calibration curve was made using ascendant concentrations (25, 50, 100, and 200 *μ*g/mL) of the isolated compounds, which were injected by triplicate at 10 *μ*L in the previously described HPLC method. A chromatographic profile of each concentration was obtained at 254 or 360 nm wavelength and data on area under curve peak were used to obtain the respective straight–line equations.

### 2.5. Enzymatic Inhibition Assays

Pancreatic lipase inhibition assay was previously reported [22]. Briefly, the absorbance of a mixture of DTNB 0.2 mM, DMPTB 0.8 mM, NaCl 0.1M, CaCl2 2 mM, Triton X–100 0.04%, porcine lipase 65 *μ*g/mL, and the sample (dissolved in DMSO and water) at 0.25 mg/mL was followed with a Thermo Scientific Genesys 20 Visible Spectrophotometer (Fisher Scientific, 4001000, Hampton, NH) at 412 nm every 20 s for five minutes and plotted (Excel, Microsoft) to obtain initial velocity value. The lipase was prepared as a stock at 10 mg/mL in Tris–HCl 25 mM pH 6.2 with 0.1 M NaCl, SDS 2 mM, and 250 *μ*L/mL of glycerol. A control assay without substrate was carried out to discard nonspecific reactions with DTMB. All reactions were tested by triplicate.

The *α*–glucosidase assay was previously reported [10]. In brief, corn starch (4 mg/mL) was digested by crude enzyme at 37°C during 10 minutes in a phosphate buffer pH 7 solution at a sample concentration of 0.6 mg/mL (dissolved in DMSO and water). Subsequently, released glucose was quantified by a glucose oxidase-based clinical reagent with the GOD–POD Trinder kit (Spinreact, Girona, Spain) following manufacturer's directions. All tests were performed in quadruplicate. Crude enzyme was obtained directly from healthy Wistar rats (12 h fasting). The small intestine was flushed several times with ice-cold isotonic buffer pH 7 and after the scraping of the mucosa, it was homogenized and stored at -20°C. Animal care and management were carried out under the guidelines of Mexican Official Standard NOM–062–ZOO–1999.

For both assays, percentage of inhibitions was calculated as the residual enzymatic activity of the negative control (DMSO and water) by using

(1) $$\begin{array}{c} \%\;inhibition=100-\left(\;\frac{{Absorbance}_{sample}}{{Absorbance}_{control}}\times 100\;\right) \end{array}$$

Concentrations of extracts resulting in 50% inhibition of enzyme activity (IC_50_ values) were determined graphically, quantifying enzymatic activities at ascendant concentrations of each sample (6–3600 *μ*g/mL for *α*–glucosidases and 5–2500 *μ*g/mL for pancreatic lipase). The logarithm of the concentration was plotted on the x-axis and the percentage of enzymatic inhibitory activity on the y-axis to obtain a semilogarithmic graphic.

The type of inhibition was determined quantifying the activity with and without inhibitor at different substrate concentrations (5–0.35 mg/mL for *α*–glucosidases and 0.05–0.2 *μ*g/mL for pancreatic lipase) and comparing Lineweaver–Burk plots (inverse substrate concentration [S] and inverse reaction velocity V). In the case of the determination of *α*–glucosidase type of inhibition, the substrate was changed from corn starch to maltodextrin (MD100, Luzhou Bio–Chem Technology Co., Shandong, China), in order to have greater uniformity in the reaction.

Michaelis–Menten constant (K_m_) and apparent K_m_ (K_m_^app^) were obtained analysing the Lineweaver–Burk plots. These values allowed to obtain the inhibition constant (K_i_) for competitive inhibitors using (2), where [I] represents inhibitor concentration.

(2) $$\begin{array}{c} {K_{m}}^{app}=K_{m}\left(\;1+\frac{\left[I\right]}{K_{i}}\;\right) \end{array}$$

### 2.6. Statistical Analysis

Experimental enzymatic inhibition activity values are expressed as the percentage of inhibition. All biological assays were analysed by ANOVA followed by a Tukey post–test, with statistical differences established at p<0.05, using the SPSS10.0 program.

## 3. Results

### 3.1. Fractionation of Hydroalcoholic Extract

The liquid–liquid separation of LoHAE produced LoAqF (82.3% yield; 156 g) and LoEAF (17.1%; 32 g). Samples of these materials and CsHAE were analysed in the* in vitro* models of enzyme inhibition at 0.6 mg/mL in the case of *α*–glucosidases and at 0.25 mg/mL in the case of pancreatic lipase (see Table 1).

LoHAE inhibited the *α*–glucosidases by 58.9% and the pancreatic lipase by 23.6%, while CsHAE produced an 80.8% inhibition of *α*–glucosidases and 34.8% of pancreatic lipase. The organic fraction, LoEAF, had more inhibitory activity than LoAqF fraction or LoHAE extract in both assays, with an 82.8% inhibition of *α*–glucosidases and 31.2% inhibition of pancreatic lipase.

**Figure 1:**
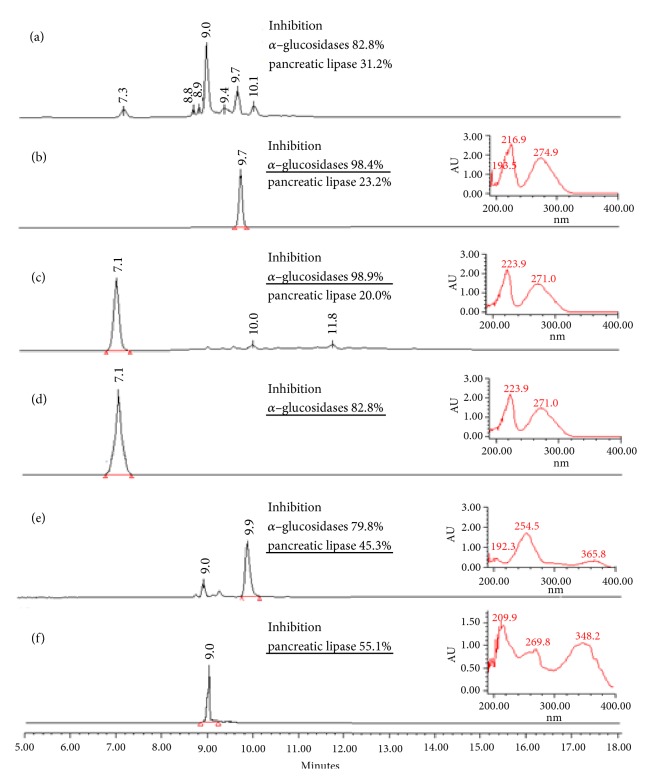
High Performance Liquid Chromatography chromatograms, UV spectra (at 270 nm), and enzymatic inhibition percentage of different* L. octovalvis* fractions. (a) Ethyl acetate fraction LoEAF. (b) Fraction C1F1. (c) Fraction C1F4. (d) Fraction C2F1. (e) Fraction C1F6. (f) Fraction C4F4–P.

High Performance Liquid Chromatography spectra analysis of LoEAF (see Figure 1(a)) indicated the presence of flavonoids and organic acids [20, 23]. The first chromatography separation of LoEAF afforded 60 fractions, which were grouped in six (C1F1–C1F6), where C1F1 and C1F4 fractions displayed the highest inhibitory effect on *α*–glucosidases, while C1F6 was the most active for lipase (see Table 1).

### 3.2. Identification of *α*–Glucosidase Inhibitors

**Figure 2:**
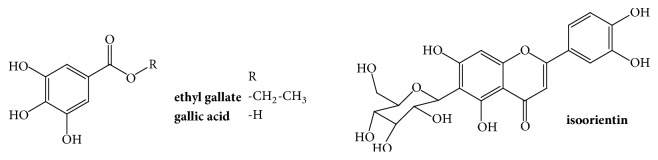
Chemical structure of the most active compounds identified in* L. octovalvis* hydroalcoholic extract.

Fraction C1F1 produced a white precipitate (melting point = 160°C) that was analysed by HPLC (see Figure 1(b)) and its chemical structure was corroborated by comparison of spectroscopic ^1^H and ^13^C NMR data (see Table 2 and Figures S1–S2 in the Supplementary Material) indicating that this compound corresponds to ethyl gallate [24] (see Figure 2).

Fraction C1F4 produced Fraction C2F1, which also produced a white precipitate (melting point= 260°C). HPLC, UV spectra (see Figures 1(c)–1(d)), and spectroscopic ^1^H NMR analysis (see Figure S3 in the Supplementary Material) indicated that this fraction corresponds to gallic acid [24] (see Figure 2).

According to HPLC analysis (see Figure S4 in the Supplementary Material), LoHAE and LoEAF contained, respectively, 0.7% and 4.6% of ethyl gallate and 1.9% and 2.5% of gallic acid.

### 3.3. Identification of Pancreatic Lipase Inhibitors

Fraction C1F6 was analysed by HPLC where several kinds of organic constituents were observed (see Figure 1(e)). Subsequent chromatographic separations of this fraction, followed by inhibitory activity evaluation (see Table 1), allowed us to obtain 11 fractions (see Scheme 1) with different chemical profiles but similar inhibitory activities. The most active fraction, C4F4–P (melting point = 245°C), was evaluated by HPLC (see Figure 1(f)) and elucidated by ^1^H NMR, ^13^C NMR, and two–dimensional NMR spectroscopy experiments (see Table 2 and Figures S5–S9 in the Supplementary Material) and corresponded to isoorientin [25] (see Figure 2). The other active fractions are constituted mainly by flavonoids and other nonidentified compounds.

According to HPLC analysis (see Figure S4 in the Supplementary Material), LoHAE and LoEAF contained 0.2% and 0.1% of isoorientin, respectively.

### 3.4. Calculating Half–Maximal Inhibitory Concentration and Determining Type of Inhibition

#### 3.4.1. *α*–Glucosidases

**Figure 3:**
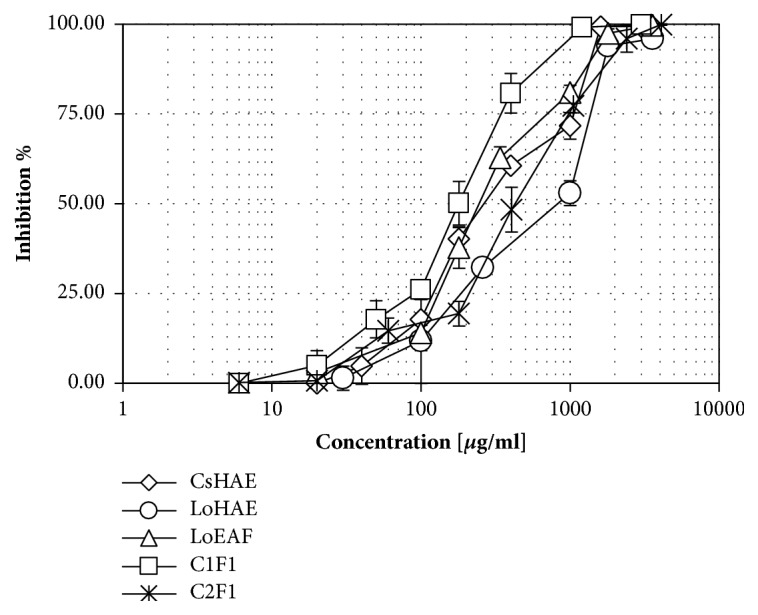
Concentration–response graphics for half–maximal Inhibitory Concentration (IC_50_) determination of CsHAE, LoHAE, LoEAF, C1F1 (isolated ethyl gallate), and C2F1 (isolated gallic acid), in the inhibition model of *α*–glucosidases. X-axis values are presented in *μ*g/mL (real values are logarithmic). The error bars represent the standard deviation of 2 measurements in four separate sample runs (n = 8).

All graphs corresponding to concentration–response curves in the *α*–glucosidase inhibition model are shown (see Figure 3). CsHAE displayed a value of half–maximal Inhibitory Concentration (IC_50_) 260 *μ*g/mL, while LoHAE produced IC_50_ 700 *μ*g/mL. Ethyl gallate (C1F1) and gallic acid (C2F1) IC_50_ values were 832 *μ*M and 969 *μ*M, respectively. Luteolin (Sigma, L9283) was used as a naturally occurring reference displaying an IC_50_ = 1257.7 *μ*M.

**Figure 4:**
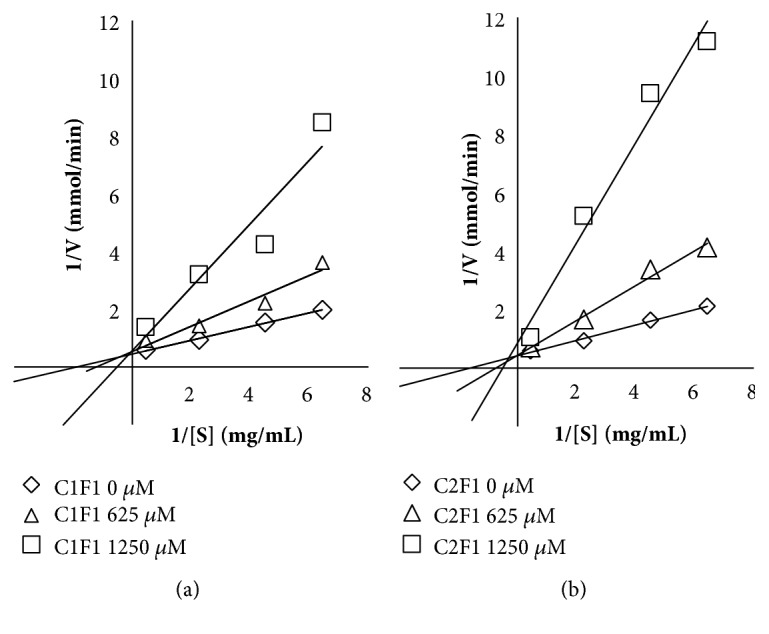
Determination of enzymatic inhibition type by Lineweaver–Burk plots curves in the *α*–glucosidase inhibition model. (a) C1F1 (isolated ethyl gallate). (b) C2F1 (isolated gallic acid).

Both compounds, ethyl gallate and gallic acid, make K_m_ (intersection x-axis) increase, but maximal velocity (V_max_; intersection y-axis) remains the same, as expected for a competitive enzymatic inhibition (see Figures 4(a)–4(b)).

For the particular conditions of this assay, the calculated K_m_ was 460 ± 3 *μ*M. In the case of K_i_ constants, for ethyl gallate at 625 *μ*M, K_i_ = 636*μ*M and at 1250 *μ*M, K_i_ = 315 *μ*M; for gallic acid at 625 *μ*M, K_i_ = 436 *μ*M and at 1250 *μ*M, K_i_ = 208 *μ*M.

#### 3.4.2. Pancreatic Lipase

**Figure 5:**
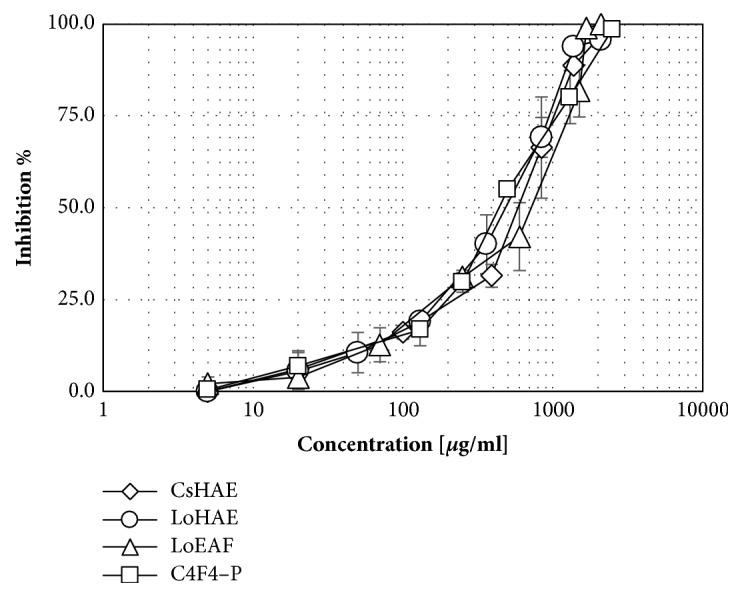
Concentration–response graphics for half–maximal Inhibitory Concentration (IC_50_) determination of CsHAE, LoHAE, LoEAF, and C4F4–P (isolated isoorientin) in the inhibition model of pancreatic lipase. x-axis values are represented in *μ*g/mL (real values are logarithmic). The error bars represent the standard deviation of 2 measurements in three separate sample runs (n = 6).

The positive vegetal control,* C. sinensis,* displayed an IC_50_ value of 587 *μ*g/mL, while LoHAE displayed 480 *μ*g/mL, LoEAF 718 *μ*g/mL, and isoorientin 201 *μ*M (see Figure 5).

**Figure 6:**
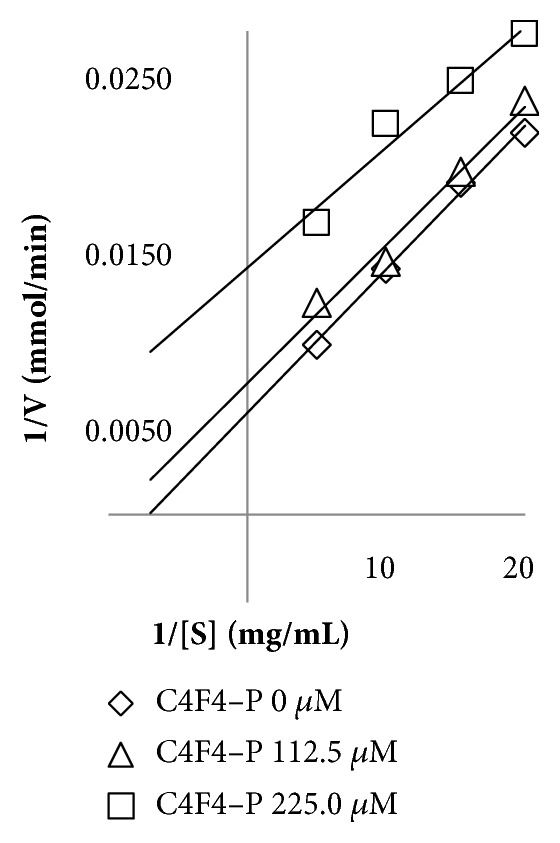
Determination of enzymatic inhibition type of C4F4–P (isolated isoorientin) by Lineweaver–Burk plots curves in the pancreatic lipase inhibition model.

As observed in the graph (see Figure 6), isoorientin changed both V_max_ and K_m_ (both intersection axes), so it produced uncompetitive enzymatic inhibition of pancreatic lipase [26].

## 4. Discussion

According to several studies, postprandial hyperglycaemia periods, even the relative short–lasting ones, contribute to the development of chronic diabetes complications even more than basal hyperglycaemia [27]. Moreover, the management of postprandial hyperglycaemia is more difficult to achieve than basal glucose control, even with a satisfactory HbA1c control [28], making it one of the main problems in diabetes treatment [1]. Of all the available antidiabetic drugs, *α*–glucosidase inhibitors are currently the most effective and safest for postprandial glycaemia control as well as intraday and interday glucose fluctuation [29]. On the other hand, changes have also been found in postprandial lipaemia and plasma free fatty acids (fasting and postprandial) in patients with type 2 diabetes mellitus, which increase macrovascular damage [30] and also may cause *β*–cell dysfunction [31]. What is worse, when high levels of free fatty acids couple with glycaemic fluctuations, they not only cause endothelium damage [32], but also have a prooxidant effect on pancreatic *β* cells, leading to *β*–cell exhaustion [33]; this phenomenon has been called glucolipotoxicity. However, it has been shown that orlistat, a lipase inhibitor, significantly improves postprandial lipaemia and free fatty acid levels in nondiabetic hyperlipidemic subjects and also in overweight type 2 diabetic patients [34, 35].

*L. octovalvis* hydroalcoholic extract has the advantage of displaying both *α*–glucosidase and pancreatic lipase inhibition activities. This is the first time that these mechanism modes are described for this species. Besides* L. octovalvis* is an interesting option as antidiabetic because it was described as innocuous according to the OECD [12].

In this study, the concentration of low and intermediate polarity compounds contained in LoEAF considerably increased the inhibition of both digestive enzymes, although an increase of *α*–glucosidase inhibition was also observed in LoAqF, indicating the presence of other polar compounds with high inhibitory activity of these enzymes. Nevertheless, according to HPLC quantitative analysis, the bipartition process successfully increased the concentration of the two *α*–glucosidase inhibitors in the organic fraction. Therefore, it would be proper to design an extraction or separation method that concentrates these polyhydroxy benzoic acid derivatives. Although gallic acid has been previously described for* L. octovalvis *[15], this is the first time that its ethyl ester derivative (ethyl gallate) is identified and related to the biological activity. The inhibition of these compounds using intestinal rat enzyme and starch as substrate was found higher than that produced by the natural product reference luteolin (IC_50_ ≈ 1257.7 *μ*M) which has been described as good inhibitor of *α*–glucosidases [36–38].

The inhibitory activity of carbohydrate degrading enzymes by gallic acid and its esters, such as ethyl gallate, has been described with inconsistent results. According to some authors, gallic acid showed very low or no inhibitory activity on porcine and Bacillus sp. *α*–amylase on rat and* Saccharomyces* sp. *α*–glucosidases on rat maltase [39–43]. However, other studies report that this compound shows high inhibitory activity on rat [42, 44] and yeast *α*–glucosidases [45] and on porcine *α*–amylase [43]. Moreover it was found that gallic acid was able to inhibit mouse, rabbit, and rat sucrose as well as rat maltase and trehalase [46]. Furthermore, the IC_50_ values of gallic acid and ethyl gallate in the inhibition of maltase (390 *μ*M, 415 *μ*M) and sucrase (130 *μ*M, 660 *μ*M) in rat were considered significantly high values [40].

This inconsistency of results could be due in part to the diversity of enzymes and substrates used for these tests; it has been shown that the effect of *α*–glucosidase inhibitors varies according to the origin of the enzymes and the type of substrate used. According to Oki* et al*. [47], to perform the best evaluation of possible *α*–glucosidase inhibitors for clinical use, mammalian enzymes and natural substrates of each type of enzyme should be used. Results of this work strengthen the hypothesis that these phenolic compounds (gallic acid and ethyl gallate) could be active in the inhibition of human *α*–glucosidases.

In this study, ethyl gallate and gallic acid displayed a competitive enzymatic inhibition, in which the inhibitor competes directly with the substrate for the binding site in the active site of the enzyme [27]. This is one of few studies in which the enzymatic inhibition type and K_i_ of naturally occurring compounds are described on digestive enzymes [48].

In the case of lipase inhibition, the most active compounds were enriched in the organic LoEAF fraction. Further purification by silica chromatography allowed us to obtain a C–glycosylated flavone: isoorientin [13]. This flavonoid displayed the best inhibitory effect and most of the fractions that produced significant activity (C1F6, C4F3, C4F5, and C4F6) contain high levels of isoorientin.

These kinds of C–glycosylated flavonoids have shown high inhibition of pancreatic lipase and according to some authors, glycosylation in position C–8 seems to significantly increase this biological activity [42–44].

Considering that it is desirable to have reference compounds to standardize a phytopharmaceutical drug, isoorientin could fulfil this purpose in* L. octovalvis* extracts with pancreatic lipase inhibitory action.

According to a toxicity analysis of this plant, an alcoholic extract from* L. octovalvis *did not display acute toxicity in mice when it was tested at 5000 mg/kg nor subacute toxicity at 400 mg/kg during 28 days [12], which is essential in the development of new phytomedicines. Furthermore, it is worth mentioning that the findings of the present study validate the traditional use of this plant species in the treatment of diabetes and also as an alternative to synthetic drugs such as acarbose and orlistat, since* L. octovalvis *displayed at least two mechanisms of antidiabetic and antiobesity action, which are synergistic and complementary.

Although none of the* L. octovalvis* treatments were as potent as the reference drugs, there are reports where* in vitro* digestive enzyme inhibition of naturally occurring compounds is lower than acarbose or orlistat but when tested on* in vivo* models, they produced similar pharmacological activities [49, 50].

## 5. Conclusions

The chemical separation of* L. octovalvis* hydroalcoholic extract which is bioactive in *α*–glucosidase and pancreatic lipase inhibition allowed the identification and pharmacological characterization of one flavone (isoorientin) with considerable inhibitory effect of pancreatic lipase and two isolated compounds with high inhibitory effect of the *α*–glucosidases (ethyl gallate and gallic acid). These findings bear out one of the possible mechanisms of action by which this medicinal plant could help in the prevention and treatment of type 2 diabetes and obesity; therefore, these data will be useful in the development of a potential novel phytomedicine.

## Figures and Tables

**Table 1:** Enzyme inhibition of hydroalcoholic extract, fractions, and compounds isolated from *L. octovalvis* leaves.

Sample	Inhibition percentage	
	*α*–glucosidases 0.6 mg/mL	Pancreatic lipase 0.25 mg/mL
Acarbose	50.0 ± 1.6*∗*	N.A.
Orlistat	N.A.	50.0 ± 2.6*∗∗*
CsHAE	80.8 ± 1.1	34.8 ± 2.5
LoHAE	58.9 ± 5.7	23.6 ± 2.5
**LoEAF**	**82.8 ± 3.6**	**31.2 ± 1.9**
LoAqF	76.8 ± 1.9	15.6 ± 2.5
**C1F1 (ethyl gallate)**	**98.4 ± 2.0**	23.2 ± 3.0
C1F2	60.1 ± 5.5	22.5 ± 3.6
C1F3	39.9 ± 5.6	4.3 ± 3.5
**C1F4**	**98.9 ± 1.6**	20.0 ± 2.3
C1F5	84.2 ± 5.3	28.2 ± 2.7
**C1F6**	79.8 ± 3.8	**45.3 ± 0.6**
**C2F1 (gallic acid)**	**98.9 ± 0.6**	N.A.
C3F1	N.A.	10.9 ± 0.3
C3F2	N.A.	29.3 ± 3.6
C3F3	N.A.	43.5 ± 4.3
C3F4	N.A.	36.4 ± 4.0
C4F1	N.A.	41.4 ± 3.2
C4F2	N.A.	16.6 ± 4.5
C4F3	N.A.	45.8 ± 5.1
**C4F4**–**P (isoorientin)**	N.A.	**55.1 ± 3.1**
C4F5	N.A.	53.5 ± 3.7
C4F6	N.A.	49.1 ± 3.8
Luteolin	66.3 **± **5.6	N.A.

The data is indicated as the mean ± standard deviation.

N.A. = not analysed; *∗* evaluated at 5.8 *µ*M; *∗∗* evaluated at 1.6 *µ*M.

**Table 2:** Nuclear Magnetic Resonance (NMR) ^13^C data of the compounds contained in C1F1 and C4F4–P fractions and previously reported data for ethyl gallate and isoorientin.

Carbon position	Chemical shifts (ppm)			
	Ethyl gallate	C1F1	Isoorientin	C4F4–P
1	121.9	121.95	–	–
2	110.1	110.18	163.44	163.61
3	146.4	146.57	102.38	102.78
4	139.6	139.79	181.45	181.84
5	146.4	146.57	160.59	160.67
6	110.1	110.18	108.88	108.86
7	168.6	168.69	163.44	163.23
8	61.6	61.81	93.73	93.46
9	14.6	14.73	156.27	156.16
10	–	–	102.79	103.38
1′	–	–	121.56	121.4
2′	–	–	118.82	118.95
3′	–	–	116.00	116.02
4′	–	–	150.44	149.68
5′	–	–	145.95	145.72
6′	–	–	112.92	113.29
1^″^	–	–	73.18	73.02
2^″^	–	–	70.50	70.60
3^″^	–	–	78.95	78.93
4^″^	–	–	70.19	70.17
5^″^	–	–	81.35	81.56
6^″^	–	–	61.34	61.48

## Data Availability

The data used to support the findings of this study are available from the corresponding author upon request.

## Supplementary Materials

Supplementary Materials Figure S1: Nuclear Magnetic Resonance (NMR) 1H spectrum of C1F1 and structure of the identified compound, ethyl gallate. Figure S2: Nuclear Magnetic Resonance (NMR) 13C spectrum of C1F1 and structure of the identified compound, ethyl gallate. Figure S3: Nuclear Magnetic Resonance (NMR) 1H spectrum of C2F1 and structure of the identified compound, gallic acid. Figure S4: calibration curves of the HPLC analysis of the isolated compounds and their straight–line equations. Figure S5: Nuclear Magnetic Resonance (NMR) 1H spectrum of C4F4–P and structure of the identified compound, isoorientin. Figure S6: Nuclear Magnetic Resonance (NMR) 13C spectrum of C4F4–P and structure of the identified compound, isoorientin. Figure S7: Correlation Spectroscopy (COSY) of C4F4–P. Figure S8: Heteronuclear Single Quantum Coherence Spectroscopy (HSQC) of C4F4–P. Figure S9: Heteronuclear Multiple Bond Correlation Spectroscopy (HMBC) of C4F4–P.
