# Supplementary material for: Identification of Digestive Enzyme Inhibitors from Ludwigia octovalvis (Jacq.) P.H.Raven
Source: Evid Based Complement Alternat Med. 2018 Jul 16;2018:8781352. doi: 10.1155/2018/8781352 (PMC6076925; doi:10.1155/2018/8781352)
Supplement: Supplementary Materials — Figure S1: Nuclear Magnetic Resonance (NMR) 1H spectrum of C1F1 and structure of the identified compound, ethyl gallate. Figure S2: Nuclear Magnetic Resonance (NMR) 13C spectrum of C1F1 and structure of the identified compound, ethyl gallate. Figure S3: Nuclear Magnetic Resonance (NMR) 1H spectrum of C2F1 and structure of the identified compound, gallic acid. Figure S4: calibration curves of the HPLC analysis of the isolated compounds and their straight–line equations. Figure S5: Nuclear Magnetic Resonance (NMR) 1H spectrum of C4F4–P and structure of the identified compound, isoorientin. Figure S6: Nuclear Magnetic Resonance (NMR) 13C spectrum of C4F4–P and structure of the identified compound, isoorientin. Figure S7: Correlation Spectroscopy (COSY) of C4F4–P. Figure S8: Heteronuclear Single Quantum Coherence Spectroscopy (HSQC) of C4F4–P. Figure S9: Heteronuclear Multiple Bond Correlation Spectroscopy (HMBC) of C4F4–P. [file 8781352.f1.pdf]

# Evidence-Based Complementary and Alternative Medicine

Supplementary Material


# Identification of digestive enzyme inhibitors from Ludwigia octovalvis (Jacq.) P.H.Raven


Dulce Morales,[1,2] Guillermo Ramirez,[2] Armando Herrera–Arellano,[1] Jaime Tortoriello,[2] Miguel Zavala,[3] and Alejandro Zamilpa[2]

[1] Facultad de Medicina, Universidad Autónoma del Estado de Morelos, Cuernavaca 62350, Mexico.
[2] Centro de Investigación Biomédica del Sur, Instituto Mexicano del Seguro Social, Xochitepec 62790, Mexico.
[3] Departamento de Sistemas Biológicos, UAM–Xochimilco, Mexico city 04960, Mexico.

Correspondence should be addressed to Alejandro Zamilpa; azamilpa_2000@yahoo.com.mx


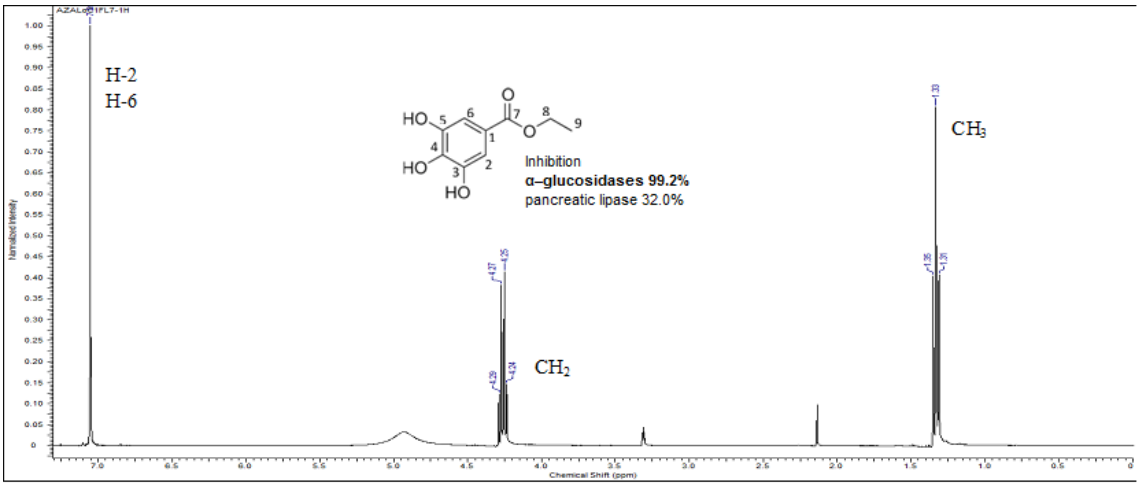


**Figure S1.** Nuclear Magnetic Resonance (NMR) $^{1}H$ spectrum of C1F1 and structure of the identified compound, ethyl gallate.

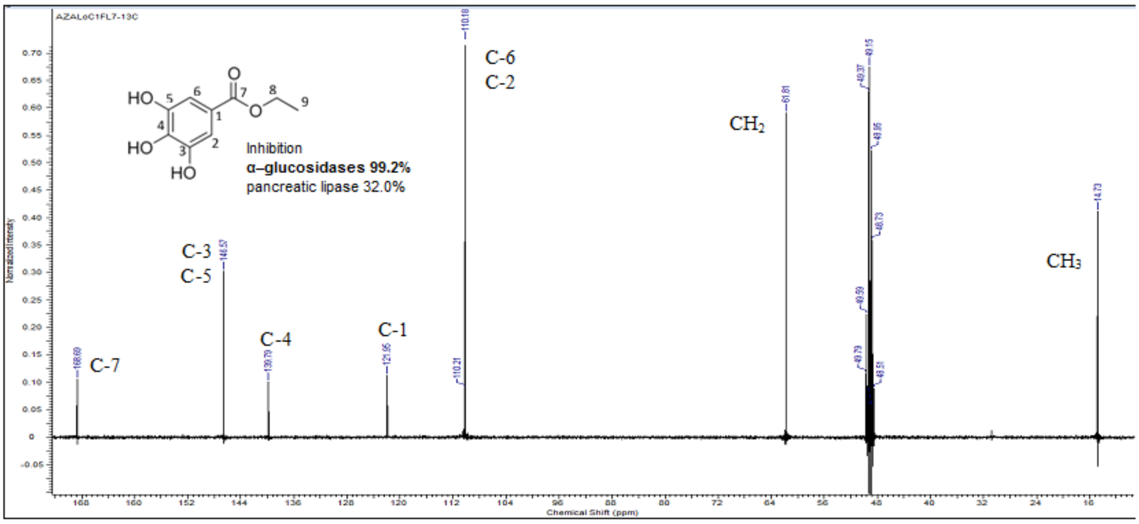


**Figure S2.** Nuclear Magnetic Resonance (NMR) $^{13}C$ spectrum of C1F1 and structure of the identified compound, ethyl gallate.

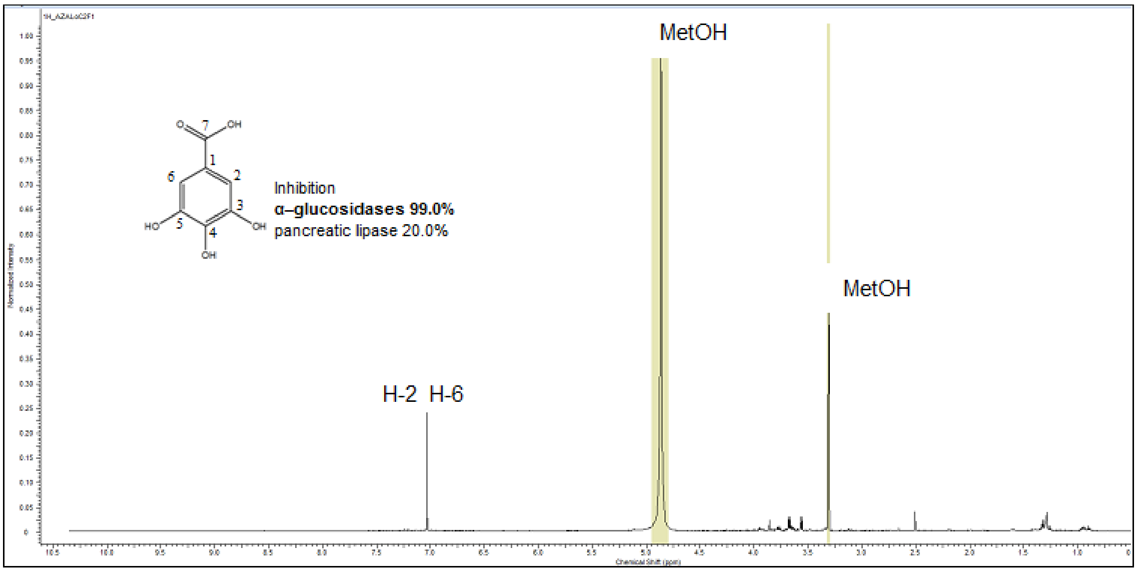


**Figure S3.** Nuclear Magnetic Resonance (NMR) $^{1}H$ spectrum of C2F1 and structure of the identified compound, gallic acid.

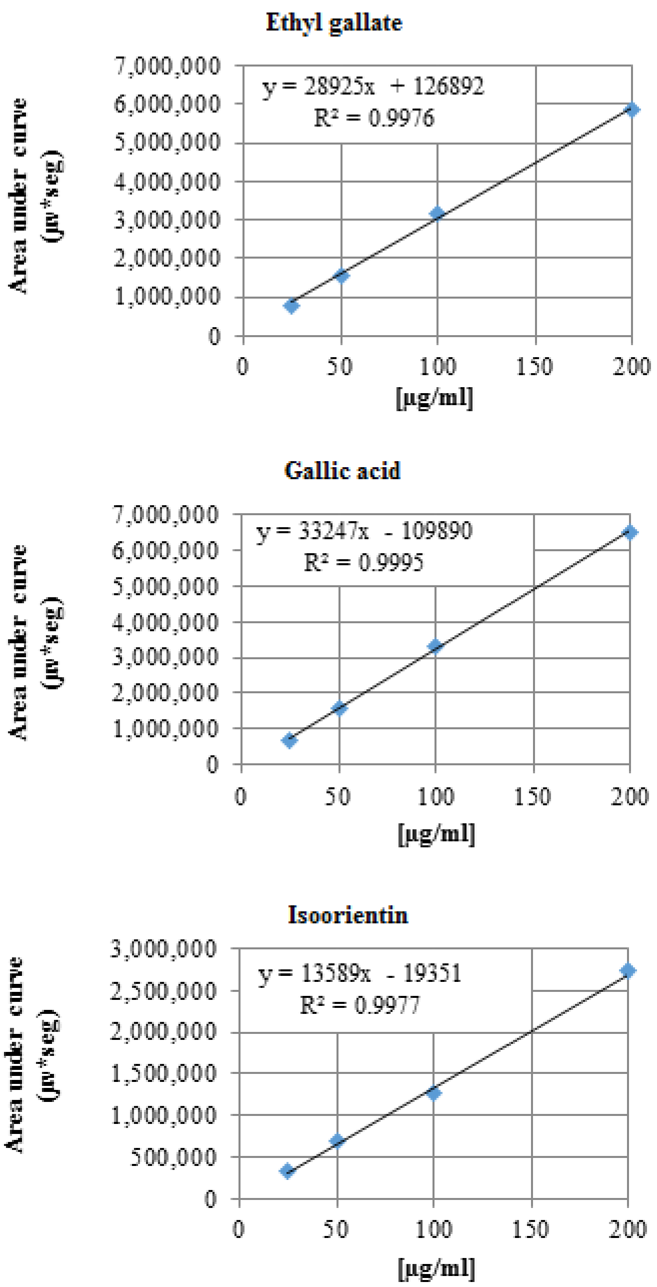


**Figure S4.** Calibration curves of the HPLC analysis of the isolated compounds and their straight-line equations.

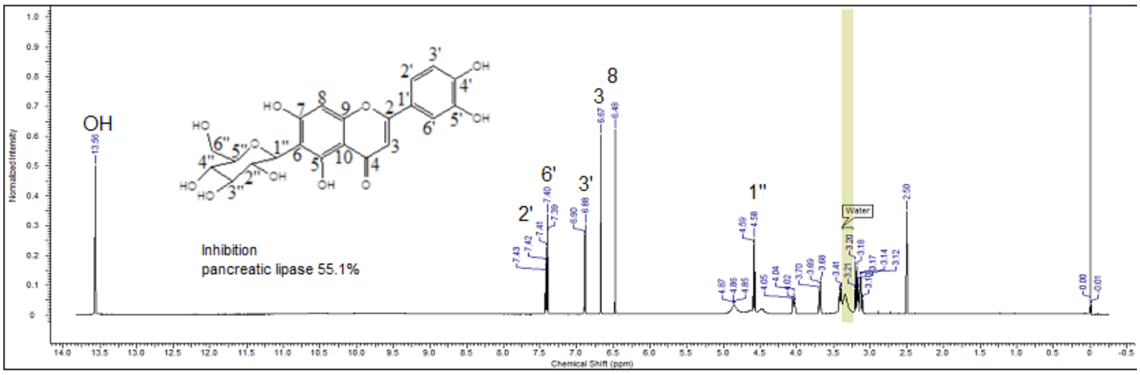

**Figure S5.** Nuclear Magnetic Resonance (NMR) $^{1}$H spectrum of C4F4–P and structure of the identified compound, isoorientin.

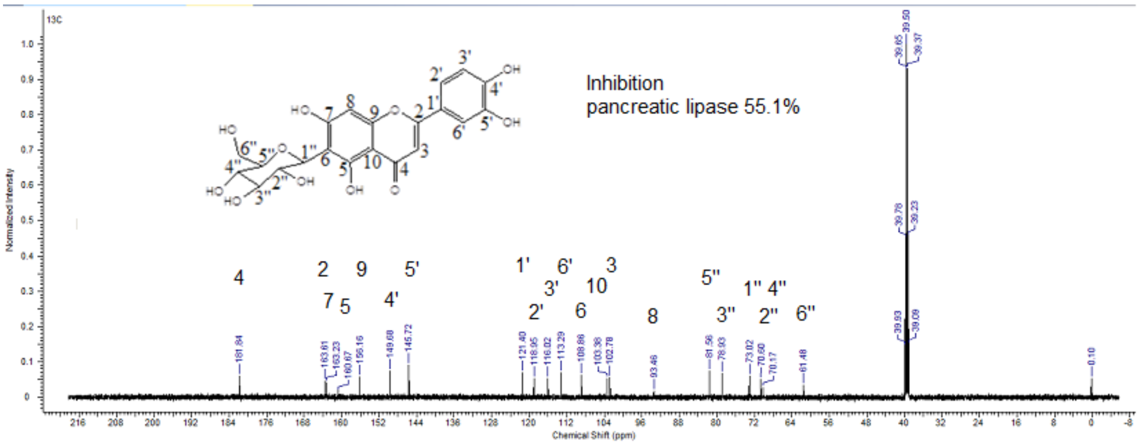

**Figure S6.** Nuclear Magnetic Resonance (NMR) $^{13}$C spectrum of C4F4–P and structure of the identified compound, isoorientin.

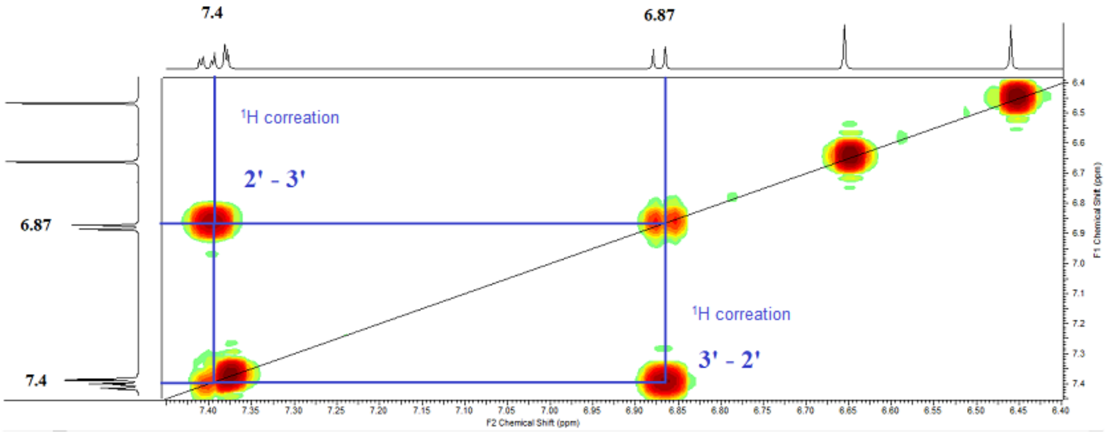

**Figure S7.** Correlation Spectroscopy (COSY) of C4F4–P.

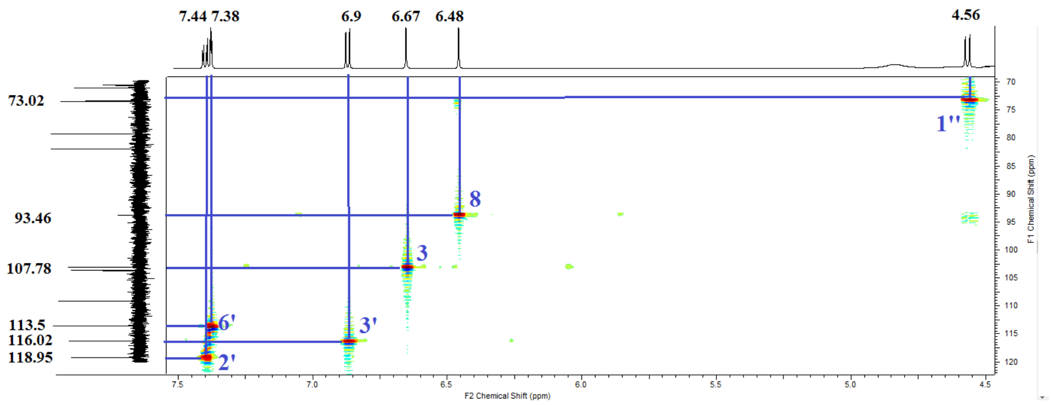


**Figure S8.** Heteronuclear Single Quantum Coherence spectroscopy (HSQC) of C4F4–P.

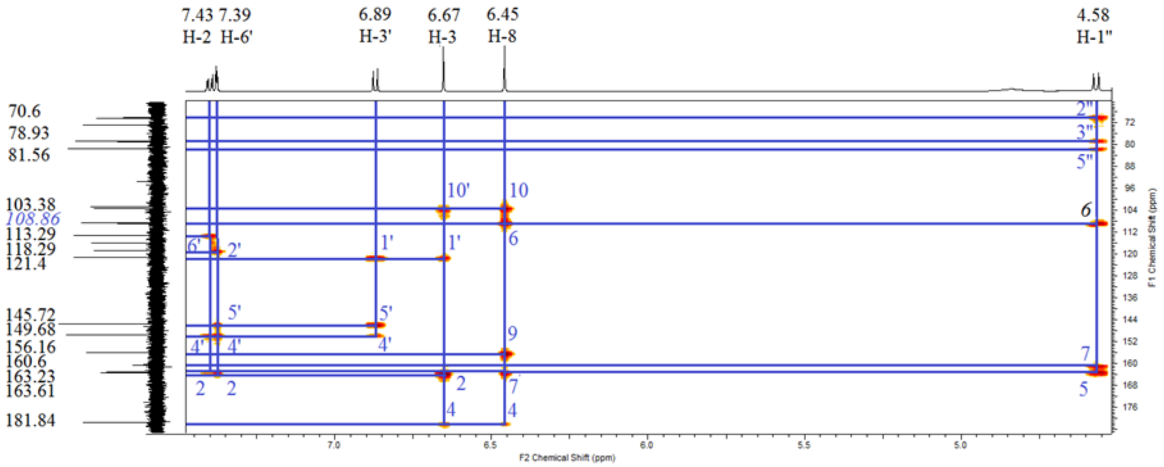


**Figure S9.** Heteronuclear Multiple Bond Correlation spectroscopy (HMBC) of C4F4–P.
